# A new phase model of the spatiotemporal relationships between three circadian oscillators in the brainstem

**DOI:** 10.1038/s41598-023-32315-y

**Published:** 2023-04-04

**Authors:** Jake Ahern, Łukasz Chrobok, Alan R. Champneys, Hugh D. Piggins

**Affiliations:** 1 School of Physiology, Pharmacology, and Neuroscience, University of Bristol, Bristol, BS8 1TD UK; 2 Engineering Mathematics, University of Bristol, Bristol, BS8 1TW UK

**Keywords:** Mathematics and computing, Applied mathematics, Neuroscience, Circadian rhythms and sleep

## Abstract

Analysis of ex vivo *Per2* bioluminescent rhythm previously recorded in the mouse dorsal vagal complex reveals a characteristic phase relationship between three distinct circadian oscillators. These signals represent core clock gene expression in the area postrema (AP), the nucleus of the solitary tract (NTS) and the ependymal cells surrounding the 4th ventricle (4Vep). Initially, the data suggests a consistent phasing in which the AP peaks first, followed shortly by the NTS, with the 4Vep peaking 8–9 h later. Wavelet analysis reveals that this pattern is not consistently maintained throughout a recording, however, the phase dynamics strongly imply that oscillator interactions are present. A simple phase model of the three oscillators is developed and it suggests that realistic phase dynamics occur between three model oscillators with coupling close to a synchronisation transition. The coupling topology suggests that the AP bidirectionally communicates phase information to the NTS and the 4Vep to synchronise the three structures. A comparison of the model with previous experimental manipulations demonstrates its feasibility to explain DVC circadian phasing. Finally, we show that simulating steadily decaying coupling improves the model’s ability to capture experimental phase dynamics.

## Introduction

Circadian rhythms are recurring biological processes with an intrinsic period of approximately 24 h. They pervade all physiology across phyla, from bacteria to mammals^1–3^ and their molecular machinery is largely conserved. Core canonical circadian clock genes and proteins function as the gears and cogs of the circadian clock^4,5^ which operate in complex feedforward and feedback loops to regulate their own transcription to produce 24 h oscillations^6^. In mammals, most cells in the brain and body contain components of the molecular clock^7,8^ and the synchronization of these molecular oscillations to the external world optimally aligns physiological functions to rhythmic environmental demands. The most predictable change in the environment is the 24-h day-night cycle, and in mammals, many circadian processes are entrained to it by the master pacemaker within the suprachiasmatic nucleus (SCN) in the hypothalamus^3,9–11^. This entrainment can be observed in rhythmic locomotor activity levels, body temperature fluctuations^12^ and circulating hormone concentrations^13^, to name a few. However, rhythmic clock gene expression occurs in other brain structures, such as the olfactory bulb^14^, the subfornical organ^15^, the habenula^16,17^, and the hippocampus^18^. Such daily transcriptomic changes can drive rhythms in neuronal activity and function of these brain regions^19–22^. From this extended neural circadian system in which extra-SCN oscillators can be distal and widely distributed^19,23^, several questions arise including (1) how this network of oscillators interact, (2) are some oscillators pacemakers and others followers, and (3) how are these oscillators synchronized to the external world. Typically these oscillators are spatially separate and may lack physical connections, so determining how oscillators interact to shape each other’s circadian timekeeping is recondite.

One way to circumvent these challenges is to focus on a brain region in which several circadian oscillators are in close physical proximity. In this regard, the dorsal vagal complex (DVC) in the medulla of the brainstem presents an accessible and tractable setting for the investigation of the principles via which multiple circadian oscillators can interact. The DVC is a key relay hub for visceral and blood-borne information which are important for a range of cardiovascular and metabolic functions^24^. It is composed of readily delineated neuroanatomical structures including the area postrema (AP), the nucleus of the solitary tract (NTS), and the dorsal motor nucleus of the vagus (DMX)^25^ as well as the cerebrospinal fluid-containing central canal/4th ventricle running along its medial axis. Rhythmic clock gene expression is reported in vivo in the AP and NTS^26–29^. Further, using a bioluminescent reporter (luciferase; LUC) of the clock protein, PER2, a recent study showed that rhythms in PER2::LUC were sustained for several days in culture in mouse DVC explants. Specifically, robust rhythms in PER2::LUC were observed in the AP and NTS as well as the ependymal cells surrounding the 4th ventricle (4Vep)^30^. Intriguingly, daily PER2::LUC expression appeared to organize in a distinct spatiotemporal order, peaking first in the AP, then the NTS, and lastly several hours later in the 4Vep.

The phase ordering between these DVC oscillators suggests there are complex relations between them, and at present, it is unclear as to how this spatiotemporal clock gene pattern in the DVC emerges or how it is sustained. Our aim with this combined modelling and experimental study is to gain an elementary understanding of physiological processes potentially responsible for this pattern of PER2::LUC phasing. Currently, we have observed a consistent phase pattern between DVC oscillators during the second day of culture recordings, which is associated with large single-cell coherence within these structures^30^. Subsequent measurements on day five show reduced coherence between cells and an altered phase difference between the AP and NTS, indicating that dynamic processes are at play. Understanding the evolution of the phase pattern may be useful for understanding the mechanisms that lead to its formation. For example, if the relationship between DVC oscillator phases arises due to rhythmic input from peripheral tissues^31^ or brain sites^32^, then in ex vivo cultures we would not expect phase relationships to be maintained. If interactions between the oscillators contribute to their phasing, then some persistence of this pattern should be observed in our recordings. There are neuronal connections between the AP and the NTS in the coronal plane^33,34^, which are both conserved in our culture preparations and exhibit a daily variation in the magnitude of their signalling^30^. Further, severing these connections significantly reduces the NTS oscillator period, whilst leaving the AP period relatively unaffected^30^, indicating a possible asymmetry in the phase communication between the nuclei.

In this study, we develop a deeper understanding of how PER2::LUC oscillations in distinct structures of the DVC change in time and we will link our previous experimental observations together into a mathematical framework capable of predicting how a DVC-like phase pattern emerges from three interacting oscillators. Our mathematical approach will be conceptual, focusing on explaining our dataset in the simplest possible terms and discovering the elementary physiological mechanisms that may be present.

## Results

### The DVC phase pattern is not consistently maintained in ex vivo slice cultures

**Figure 1:**
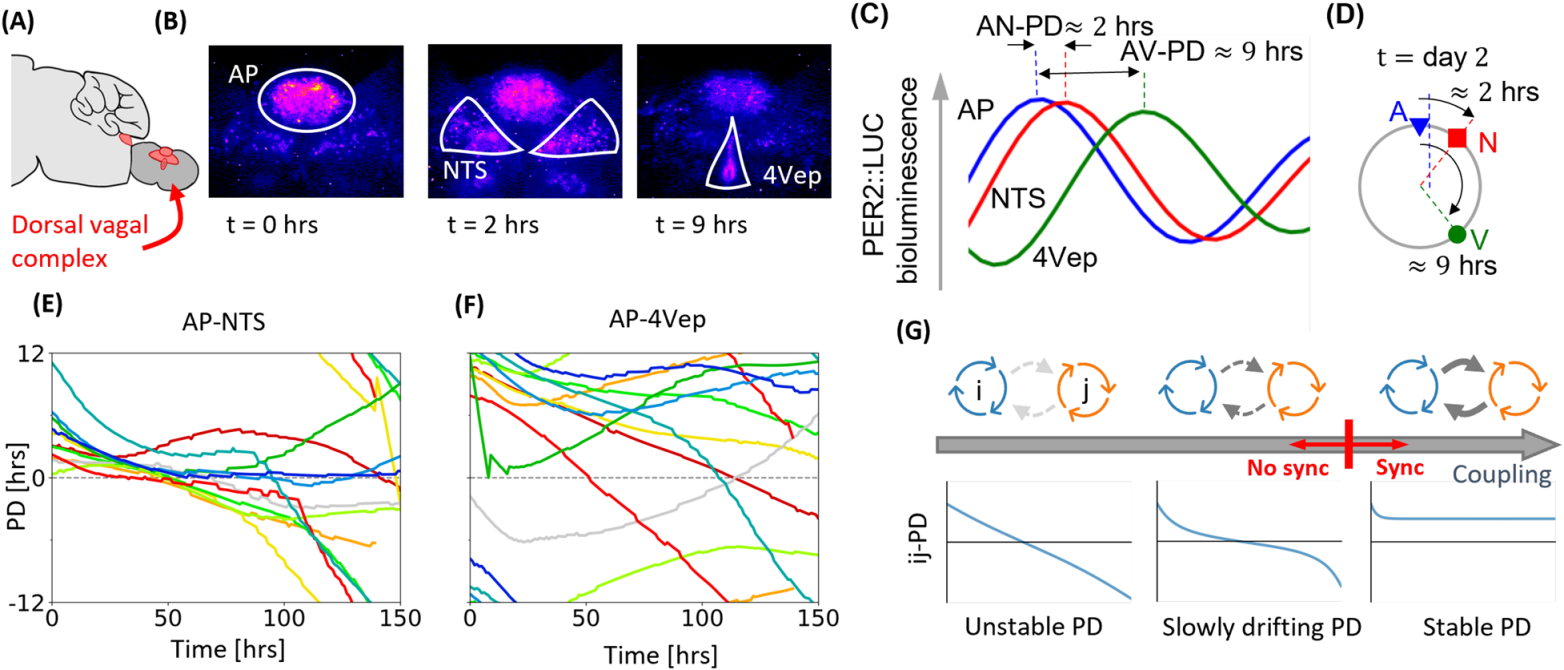
Phase difference dynamics between circadian oscillators in ex vivo DVC cultures. (**A**) The DVC, highlighted in red, in the medulla of the brainstem, initially in the sagittal plane, and then in the coronal plane. (**B**) PER2::LUC bioluminescent images from coronal sections of the DVC, taken from three different time points. (**C**) Schematic diagram of the rhythmic bioluminescent signal from the regions defined in (**B**), where the two NTS are taken as a single oscillator. (**D**) Phase relationship on day 2 of the recording (described in^30^). The PD dynamics between the AP and NTS (**E**) and the AP and 4Vep (**F**) were calculated via wavelet analysis of PER2::LUC signals. The y-axis is periodic, meaning that traces disappear from one end and reappear on the other. (**G**) If the coupling between two oscillators *i* and *j* is sufficiently small, they will not synchronise and their phase difference (PD) will drift (left). For very strong coupling, oscillator periods align and a constant PD is maintained. For intermediate coupling near the synchrony threshold (red line), the oscillator’s PD is slowly drifting (middle).

The phase difference (PD) relationship we reported in our earlier exclusively experimental study^30^, which is summarised in Fig. 1C,D, consists of a small positive AP-NTS phase difference (AN-PD) of approximately 2 h, and a large positive AP-4Vep phase difference (AV-PD) of around 9 h. Positive values indicate that the AP zenith occurs before the peak of the other oscillator. We calculated this phase relationship using one measurement on the second day of the recording (see^30^). To extend this description and gain insight into the dynamic stability of the DVC relationship, wavelet decomposition^35^ of nulcei-wide PER2::LUC signals was used to understand how the rhythmic properties change over time.

The (PD) dynamics of a combination of 11 previously and newly generated recordings of coronal DVC slice cultures are shown in Fig. 1E,F. Between the three oscillators, two PDs are plotted: the AN-PD (E) and the AV-PD (F). Only two PDs are required to describe the full phase relationship and we choose the AP as the reference oscillator because a near synchronous state in the system almost always includes the AP. Our results indicate that the PD relationship initially observed is not sustained. Specifically, in (E), there is an initial large positive PD in the first 18 h which then gradually declines throughout the subsequent 130 h such the PD between the AP-NTS eventually becomes negative in most (9/11) recordings, but can also return to positive or near positive values (2/11 cultures). This indicates that the AP initially phase leads the NTS, but that this order cannot be sustained. In (F), the AP consistently leads the 4Vep in most recordings (7/11), but in some cultures, the PD is reduced over time so that the AP lags behind the 4Vep. Note that our new analysis captures the result of our previous analysis (cf. Fig. 1D and^30^) because between 12 and 36 h, the mean phase difference of the oscillators in our original data set is calculated as 2 h for the AN-PD and 8.4 h for the AV-PD. Since the PD in the initial 24 h in culture is currently interpeted as representing the PD in vivo, this suggests that the AP does phase-lead the NTS and 4Vep, but the mechanism(s) underlying this phase arrangement is attenuated ex vivo.

While the PD relationship is generally not maintained, we also observed PD time series ranging from unstable (red plots in Fig. 1F) to stable (dark blue plot in Fig. 1E), with a majority of PDs intermediate between stable and unstable (see the “Methods” and Supplementary Fig. S5 for further details regarding the analysis of stability). The theory of weakly coupled oscillators describes this variety in PD trajectories using the notion of varying oscillator interactions (Fig. 1G). For two oscillators of different intrinsic periods and negligible interactions, their PD will be unstable and continuously grow in time. Significant interactions lead to an occasional reduction in the differences in their circadian periods, which manifests as gradually drifting PDs. Further increasing oscillator interactions leads to synchrony, where frequencies match and a constant PD is established. With this in mind, the first 100 h of our data bear similarity to an oscillator system in various states of coupling. Motivated by this, we subsequently focused on developing and analysing a simple model to investigate the possibility that sub-critical dynamics between three weakly coupled oscillators account for the PDs observed among DVC oscillators.

### Minimal Kuramoto-like model is consistent with ex vivo data

The mathematical description of the DVC oscillator network is based on a Kuramoto model^36^, in which the phase of each oscillator, $\theta _i$, where $i\in \{a,v,n \}$ for AP, 4Vep and NTS respectively, is described by an ordinary differential equation (ODE). The full system of phase evolution equations are described by (2) in the “Methods” section, and our treatment of the NTS as a single oscillator is explained in the Supplementary Information. The phase dynamics are not of direct interest to us here, and consequently we make a coordinate transform to describe the phase differences between the AP and NTS ($\theta _{an} = \theta _a - \theta _n$) and the AP and 4Vep ($\theta _{av} = \theta _a -\theta _v$). The resulting system is given by

1 $$\begin{aligned} \begin{aligned} \dot{\theta }_{an}&= \omega _{an} - \tilde{K}_{an}\sin (\theta _{an}-\gamma ) -K_{av}\sin \theta _{av} \\ \dot{\theta }_{av}&= \omega _{av} - \tilde{K}_{av}\sin \theta _{av} - K_{an}\sin (\theta _{an}-\gamma ). \end{aligned} \end{aligned}$$

Here, the parameters $\omega _{ij}=\omega _i-\omega _j$ are the frequency detuning between oscillators *i* and *j*; that is the differences between the intrinsic frequencies (related to the intrinsic period by $\omega _i = 2\pi /\tau _i$). The coupling constants $K_{ij}$ are the scalar parameters that describes the influence that oscillator *j* has upon oscillator *i*, and $\tilde{K}_{ij}=K_{ij}+K_{ji}$. Note that we have assumed that 4Vep-NTS interactions are negligible and so the appropriate coupling strengths have been set to zero ($K_{nv}=K_{vn}=0$). The justification for this assumption is that the stability of the NTS-4Vep PD is rarely observed (2/11 cultures), unless it is mediated by the AP. Furthermore, a detailed bifurcation analysis (details not shown) indicates that these parameters are inconsequential. The AN phase lag parameter, $\gamma$, tunes the AN-PD and it is essential to obtain negative steady-state AN-PD values. More information about these parameters, and the full derivation of Eq. (1), can be found in the “Methods” section and the Supplementary Information. We wish to understand what set of parameters, if any, are consistent with the phase difference trajectories we presented in Fig. 1.

#### Identifying model parameters

**Figure 2:**
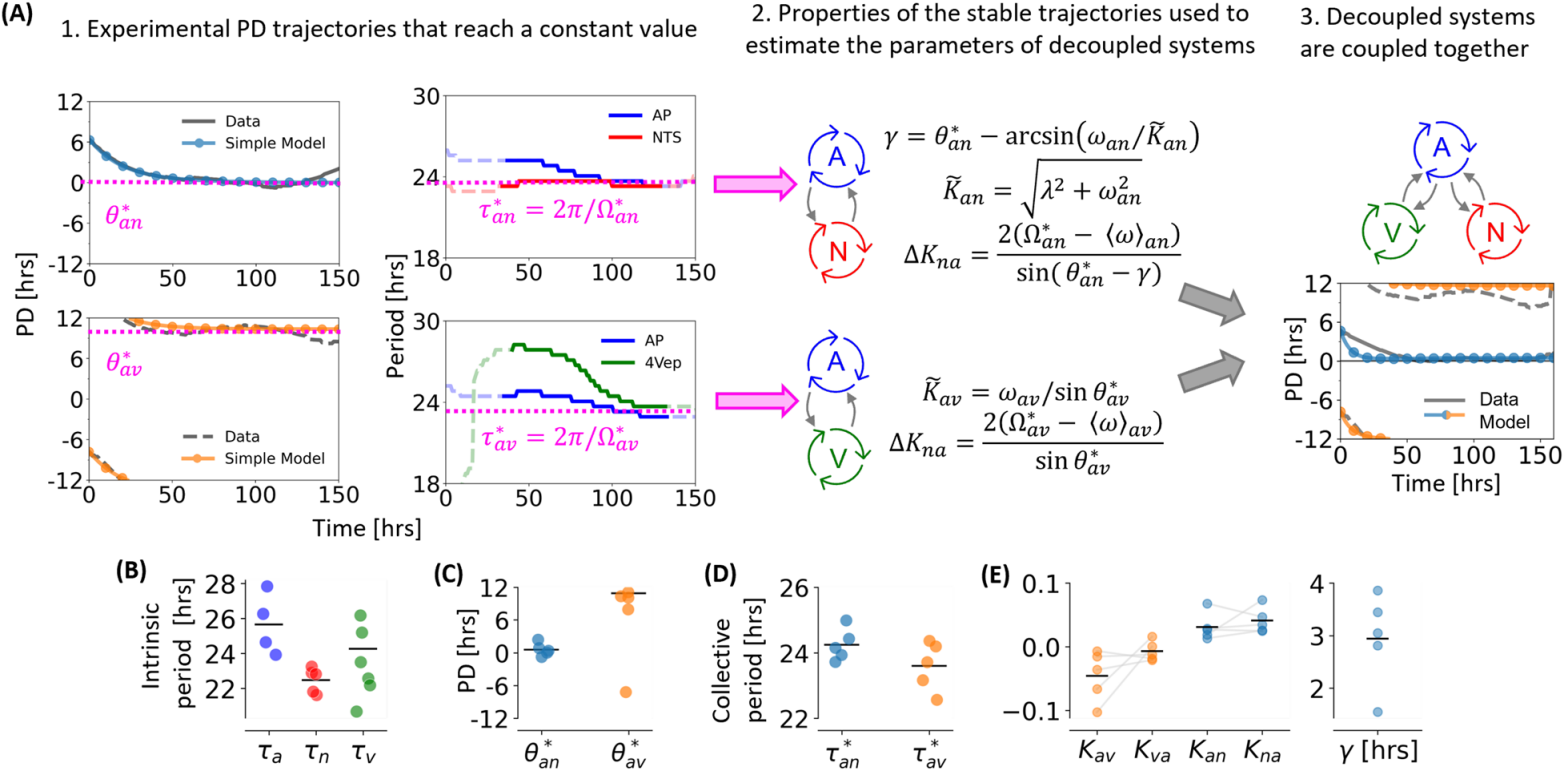
Estimating model parameters using the most stable PD recordings. (**A**) Constant PD trajectories can be analysed to find the steady state PD ($\theta _{ij}^*$) and the collective period of the two oscillators when their PD is constant ($\tau _{ij}^*$), which is used to estimate the parameters of two two-oscillator models. Recoupling the two systems creates a three-oscillator model that closely matches the data. (**B**) Intrinsic period parameters. Note that $\tau _a$ and $\tau _v$ overlap. (**C**) The constant PD between the most stable oscillator pair and (**D**) the corresponding collective periods. (**E**) The coupling parameters and AN phase lag are estimated from the most stable PD signals.

A selection of PD trajectories that remain constant for over 24 h are used to estimate the parameters in the model above, the mean values of which are presented in Table 1. To simplify the estimation process, the model is decoupled into two subsystems (AP-NTS and AP-4Vep systems) and each is fit to data separately. Figure 2A illustrates this process. The derivation of the equations in 2A and a more detailed description of how constant PD states are analysed can be found in the “Methods” section. Once the AP-NTS and AP-4Vep models are fitted, they can be coupled together into a three-oscillator system. This re-coupling results in a slightly worse fit of the three-oscillator model to the data, but such small changes in the final steady-state PDs were found not to be important for the purpose of the model.

**Table 1:** Intrinsic oscillator periods estimated from experimental data and fitted model parameters of (1) for the fully syncrhonised AP-NTS-4Vep oscillations, and their standard deviations (STD).

Parameter	Mean value	STD
${\tau _a}$	25.7 h	1.75 h
${\tau _n}$	22.5 h	0.72 h
${\tau _v}$	23.4 h	2.03 h
$K_{an}$	0.031	0.021
$K_{na}$	0.041	0.020
$\gamma$	0.770 rad	0.23 rad
$K_{av}$	− 0.045	0.039
$K_{va}$	− 0.007	0.015

The intrinsic periods of each oscillator were estimated directly from experimental data. For the AP, we calculate this period as the time-averaged period of PER2::LUC bioluminescent signals from surgically isolated and spatially separated AP explants ($n=4$; Fig. 2B), which have an average period of $25.7\pm 1.75$ h (mean ± SD). We calculate the NTS intrinsic period in a similar manner, where now one bilateral NTS is surgically separated (see Fig. 4F and^30^ for details), which has period $22.5\pm 0.72$ h ($n=5$; Fig. 2B). Notably, there is no overlap between the AP and NTS intrinsic period measures. Surgically separating the 4Vep from the surrounding oscillators is technically challenging, hence we estimate the intrinsic period as the 4Vep period in experiments where the AV-PD is least stable, which has a mean of $23.4 \pm 2.0$ h ($n=6$; Fig. 2B). Notice here that there is considerable overlap between the AP and 4Vep intrinsic frequencies.

The numerical values of the constant PD states that occur between the AP-NTS (blue) and AP-4Vep (orange) are plotted in Fig. 2C. For the AP-NTS, a small PD around zero ($0.55\pm 1.06$ h) is (semi) stable, and for the AP-4Vep, constant PDs arise at higher values ($10.9\pm 2.95$ h). In one case, the constant AV-PD is negative. The period of each oscillator during a constant PD state is calculated and averaged in time. The difference in period between two oscillators whilst their PD is constant is always less than 4% (Supplementary Figs. S3 and S4) and so the collective period of the coupled oscillators is defined as the average of the two individual periods. The collective periods of the coupled oscillators when their PD is constant ($\tau _{an}^{*}$ and $\tau _{an}^{*}$) are displayed in Fig. 2D. Each period is a compromise between the intrinsic periods of the two participating oscillators. The intrinsic periods, constant PD states and their corresponding collective periods are used to estimate model parameters, which are plotted in Fig. 2E. Model intrinsic period parameters are averages of those calculated in Fig. 2B.

#### Analysis of AP-NTS-4Vep coupled oscillator model

**Figure 3:**
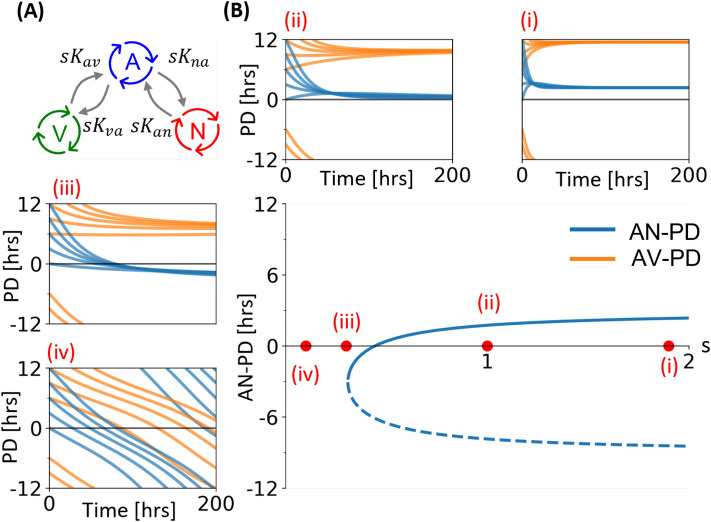
Simulated PD dynamics change as the global coupling parameter decreases. (**A**) The structure of the AP-NTS-4Vep oscillator network with the AP at the center. (**B**) Bifurcation diagram showing how the steady state $\theta _{an}^*$ changes as the global coupling strength changes. Solid lines are stable solutions and dotted lines are unstable solutions. Inserts (i-iv) show the AN-PD (blue) and AV-PD (orange) dynamics for different values of global coupling, *s*. Parameters for all the simulations are as given in Table 1, the only difference between simulations being the chosen initial conditions.

Starting from the fully synchronised AP-NTS-4Vep system using the parameter values in Table 1, we examine the effect of reducing the coupling by introducing a global coupling parameter *s* such that $K_{ij} \rightarrow s K_{ij}$, for all *i*, *j*. Reducing *s* is analogous to reducing all of the coupling parameters by the same fraction, and the result of varying the overall coupling strength is seen in Fig. 3. For strong coupling (insert (i)) the system quickly decays from its initial conditions to its steady state. The bifurcation diagram shows that as the coupling decreases, the steady state for both $\theta _{an}$ and $\theta _{av}$ decrease along a parabolic curve. Associated with lower coupling strength is a decrease in decay rate (insert (ii)). The model has a fold bifurcation close to $s=0.3$, below which the synchronous state does not exist. Trajectories for the simulated AN- and AV-PD’s have initial transients which give way to drifting solutions, with the slope of the drift increasing with lower *s* (inserts (iii) and (iv)). Our experimentally obtained PD dynamics (Fig. 1E,F and Supplementary Fig. S1) are qualitatively similar to the dynamics of the system around the bifurcation point, indicating that the ex vivo dynamics of the DVC system is close to a synchronisation transition.

### PER2::LUC data is explained by decaying coupling between nuclei

**Figure 4:**
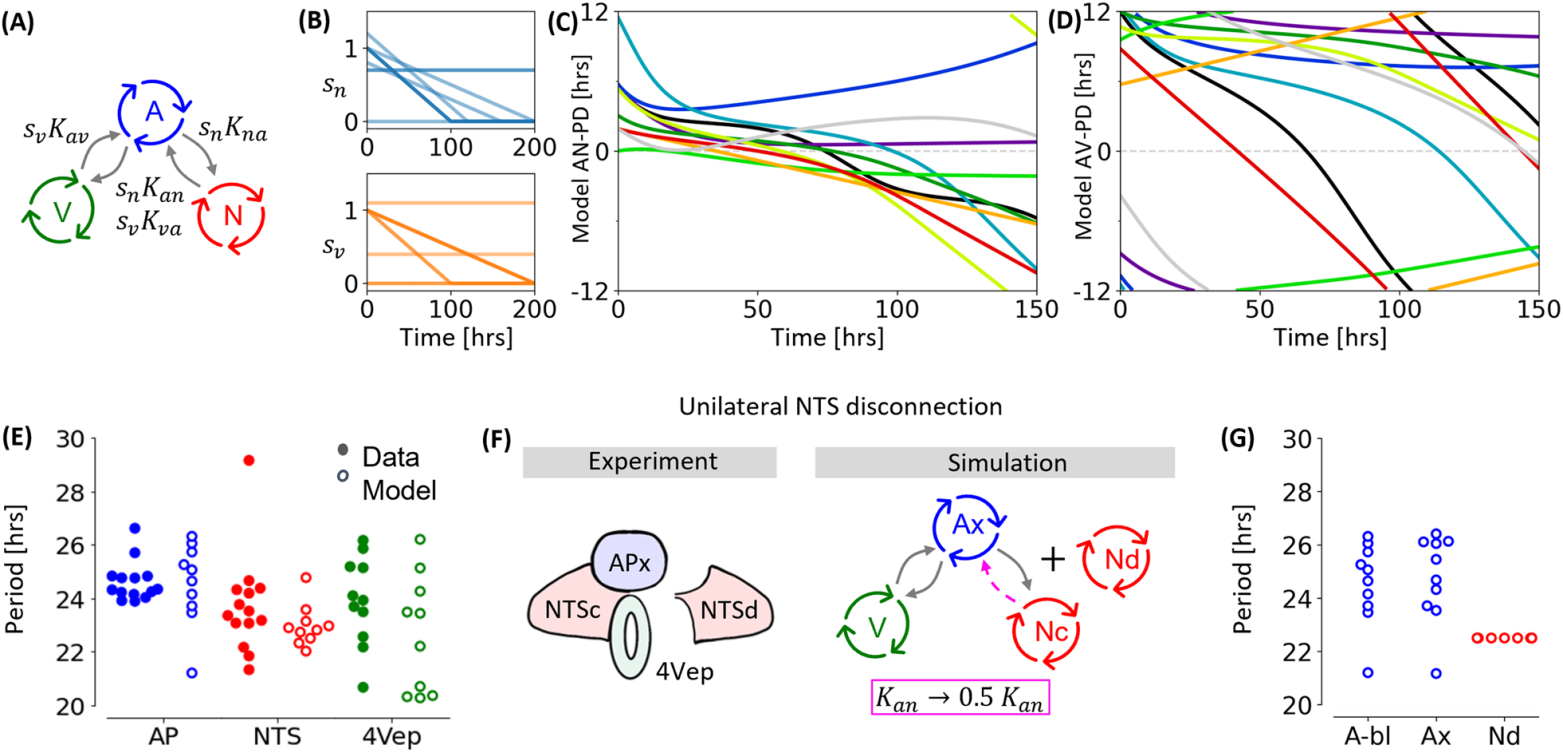
AP-NTS-4Vep coupled oscillator with decaying coupling parameters simulates multiple features of the data. (**A**) Network topology of the model. Coupling control parameters $s_n(t)$ and $s_v(t)$ are specific for AP-NTS and AP-4Vep interactions respectively. (**B**) Time course of the global coupling parameters. (**C**) and (**D**): Simulated AN-PD and AV-PD traces, respectively, with initial conditions taken from the data. (**E**) Periods of the three biological and simulated oscillators across a baseline experiment or simulation. (**F**) Schematic illustration of the surgical disconnection experiment performed in^30^ and its in silico analogue. (**G**) Results of the simulated NTS disconnection experiment for oscillators observed periodicity.

A significant difference between the ANV model and our data is that the experimental PDs occasionally show a rapid deviation from the constant or drifting state (Fig. 1E,F). Also, the model does not capture the apparent loss of synchrony that occurs following 60+ h in culture (for example, see experiments 5, 7 and 12 in Supplementary Fig. S1). This behaviour resembles the phase slip observed between two nearly synchronised oscillators, but our simulations of this scenario could not replicate the rapid nature of these slips. A reasonable hypothesis is that the coupling between the oscillators is gradually declining in the ex vivo slice. To investigate this, we introduce a time dependence into the global coupling parameter. Further, because AP-4Vep and AP-NTS communication mechanisms are likely different, on account of the 4Vep being a non-neuronal structure, we introduce two separate time-dependent coupling control parameters, $s_n(t)$ and $s_v(t)$, to describe how these interaction mechanisms may change in time (Fig. 4A).

In Fig. 4 we simulate an ensemble of AP-NTS-4Vep oscillator systems with varying global coupling decays and intrinsic frequencies. The global coupling parameters are either constant or linearly decaying, with various initial values and decay rates (Fig. 4B). Mathematically this corresponds to $s_i(t) = \max (s_{0,i} - c_it, 0)$. An initial value of $s_{0,i}=s_i(0)=1$ corresponds to coupling strengths in Table 1. Different initial values are chosen to encompass the variability we observe in the initial stability of the PD dynamics. For example, both PDs in experiment 11 (Supplementary Fig. S1) quickly become unstable and we use $s_n(0)=s_v(0)=0$ to simulate this. Some of the PD data is similar to the sub-critical dynamics of Fig. 3 (iv), and we use constant global coupling parameters at various values to simulate this behaviour. To simulate PD data that initially seems stable but subsequently drifts, a constant decay is introduced into the global coupling parameters. To achieve a qualitative likeness to the experimental data we use linear decays that decline to zero coupling between 100 and 200 h. Exponential decays of the overall coupling were also simulated, but the resulting PD dynamics were not noticeably different from a linear decay. To incorporate the natural variation of the oscillator’s intrinsic frequencies, we have also varied the intrinsic frequencies of the model AP and 4Vep oscillators. The NTS intrinsic frequency calculations (Fig. 2A) are considerably less variable than those for the AP and 4Vep, so this parameter was always set to the average value.

By varying the parameters described above we were able to qualitatively match the model to most of the individual PD dynamics, which are plotted in Fig. 4C,E. These simulations capture the dynamics that are persistently stable throughout the recording, as well as those that instantly drift. More complicated phase evolutions, including an increasing AN-PD (Fig. 4C; dark blue curve), nearly constant PDs followed by rapid drifts (Fig. 4C; teal curve) and multiple near constant regions in the same time series (Fig. 4C; black curve) are also simulated. A full likeness of any simulation to its corresponding experimental PD was not the objective of our simulations, so we have not rigorously estimated the model parameters. Instead, we aim to capture the general trend of the phase difference dynamics between DVC oscillators. In this respect, Fig. 4C,D compare well to Fig. 1E,F.

Further comparisons between the model and the data can be made by assessing the average period throughout the recording (or simulation) time (Fig. 4E). We observe that simulation periods match well with the periods calculated from the data. In particular, note that even without varying the NTS intrinsic frequency parameter, a realistic variation in the observed period is simulated. Our model can also be tested against an experiment performed in^30^ in which one of the bilateral NTS is surgically disconnected from the surrounding DVC (Fig. 4F). We previously observed that this intervention leads to the disconnected NTS (NTSd) oscillating much faster than the connected NTS (NTSc), whilst the AP (APx) frequency is not significantly altered. Here, we simulate the NTSd using a completely uncoupled NTS oscillator. The APx-NTSc-4Vep system is simulated by taking the model in Fig. 4A and reducing the NTS to AP connectivity by one half ($K_{an}\rightarrow \frac{1}{2}K_{an}$; the global coupling parameters, $s_i$ are omitted). The mathematical justification for this can be found in the Supplementary Information. The effect of this coupling reduction is shown in Fig. 4G. The period of the AP with one of the two NTS removed is similar to the period of the AP when full NTS connectivity is present. As expected, the period of the NTSd is a single value: the NTS intrinsic period, since this parameter is not varied and no other oscillators influence its dynamics.

## Discussion

### Instability of the phase relationship and its implications for the DVC and other clock networks

In our previous exclusively experimental study, we observed a spatiotemporal pattern in gene expression rhythms between three distinct regions of the hindbrain: the AP, the NTS and the 4Vep^30^. The pattern formed by this triad of oscillators is a simple phase ordering where the AP phase leads the three oscillators, with the NTS following approximately 2 h behind and the 4Vep in almost antiphase to the others, reaching its zenith around 9 h after the AP (Fig. 1B,C). If this pattern persisted throughout a recording, in which the DVC network is removed from other potentially rhythmic inputs, it can be concluded that the network itself establishes the pattern. Conversely, if the pattern is only maintained during the initial few days of recording, it would indicate that in vivo processes are required and the pattern we observe reflects its state in the intact animal. To gain insight into the dynamics of the phase pattern, we used wavelet analysis^35^ to obtain high-resolution PD (and period) dynamics. Our results show that the phase pattern is neither completely stable nor unstable ex vivo. In some cases, the PDs that define the pattern are remarkably stable, and in other cases, they are unstable (Supplementary Fig. S5). An initial inspection would imply that the DVC circadian properties are maintained via a combination of inter-network communication and external rhythmic inputs. External rhythmic input potentially shapes the DVC network phasing since the NTS is innervated by brain structures such as the lateral hypothalamus and the arcuate nucleus of the hypothalamus^24^, that are also rhythmic in PER2 expression^23,37^, as well as peripheral oscillators in the gut and nodose ganglia^31,38^. However, we argue that the dynamics observed can be effectively explained by three interacting oscillators, where their connectivity is reduced compared to in vivo conditions and is slightly variable between tissue preparations.

We demonstrate that the DVC phase dynamics can occur from the interactions of coupled oscillators using a phase model with an experimentally derived network topology. This model leads to similar phase dynamics, observable periods, and response to NTS disconnection when compared with the biological system (Fig. 4). The range of PD evolutions (Supplementary Fig. S1) is explained by two factors: firstly, the couplings between DVC nuclei are close to a synchrony threshold, and secondly, these coupling mechanisms diminish over time. In Fig. 3 we show that as global network coupling reduces below a fold bifurcation, steady states give way to PD trajectories with almost invariant plateaus. Further reducing the coupling results in rapidly changing phase differences. Mostly our data resemble an oscillator system near this synchronisation transition, which alludes to the possibility that in the absence of external inputs, the three oscillators can maintain stable phase relationships. Experiment 3 (Supplementary Fig. S1) is a potential realisation of this. Unsurprisingly, the cultured oscillators may have weakened connectivity since their preparation into thin (250 μm thick) slices can remove neural projections, thereby weakening at least one form of inter-oscillator communication^30^. Small differences in the angle of tissue slicing may be a source of variation in the global coupling that we observe. Circadian oscillators can also exert their influence using paracrine mechanisms^39,40^, as is suggested by the influence of circadian oscillations in the choroid plexus of the 3rd ventricle on the period of the SCN^41^. Paracrine signalling may be utilized between the non-neuronal 4Vep and the AP, which is more densely populated with glial cells than the NTS^42,43^, and a candidate signalling factor is transforming growth factor-$\beta$ (TGF-$\beta$) which has recently been shown to mediate peripheral oscillator interactions^44^. If paracrine signalling is utilized, this communication pathway will be significantly weakened or absent in our experiments due to the relatively large volume of culture medium compared to the size of the explant. It is worth noting that in this study we have only considered how oscillator coupling and intrinsic frequency differences affect phasing. Other properties such as oscillation amplitude, intercellular coupling strength and the aftereffects of in vivo zeitgebers^45–47^ may also determine DVC phasing, and future studies should consider these effects.

It is possible then that the system we have recorded from is in a state of reduced connectivity, and that in vivo there is sufficient coupling between the oscillators for synchronisation and a persistent phase pattern. Our model would suggest that stronger coupling is associated with larger phase separations between the NTS/4Vep and the AP (Fig. 3). This effect can be observed with methods used in vivo to quantify clock components in the AP and NTS^28,30^, which have a PD close to a few hours. Subcritical coupling may also be biologically functional. Previous studies^48–51^ indicate that subthreshold coupling facilitates a more flexible oscillator system, capable of responding to a wide range of cues. The DVC is a site of significant information integration from central and peripheral areas, some of which are rhythmic. It may be advantageous for oscillator phasing to be altered by external cues such that the network PDs can encode relevant information.

The DVC phase dynamics is captured best when the global coupling parameter is separated into an AP-NTS coupling, $s_n$, and an AP-4Vep coupling, $s_v$, both of which can decay over time (Fig. 4A,B). A gradual time decay allows the system to exhibit synchronous (or near-synchronous) behaviour before showing rapidly changing PDs, indicative of very weak coupling. Experiments 5, 8 and 11 are examples of such behaviour. In experiment 7, rapid reduction in $s_n$ compared to $s_v$ leads to the AP synchronising to the 4Vep and becoming faster than NTS, leading to an increasing AN-PD (Supplementary Fig. S1). Other dynamic profiles that are readily simulated using a decaying global coupling, but not static couplings, include the AN-PD traces in experiments 9 and 11. Decreasing coupling likely reflects a deterioration in tissue integrity. Even in the SCN, it is well known that single-cell autonomous circadian oscillations desynchronise over time, likely due to a reduction in cellular communication^52,53^, which we have also observed between single cells of the AP and NTS^30^. To gain a better understanding of the coupling decay process, our phase model could be extended to the single-cell level, where phases of individual cells are modelled. The desynchrony observed between cells within DVC structures could then be incorporated into a model to describe how AP-NTS coupling, for example, deteriorates due to this reduced cellular communication between the AP and NTS.

We have compared our biological system to a deterministic mathematical system, however, biological systems are subject to noise. We have not included the effect of noise in our model, but it is well-known that noise tends to smear the synchrony transition, making a bifurcation point impossible to calculate^54^. Our data is likely displaying such behaviour.

### Model parameters and their implication for the DVC

The coupling parameters calculated between the AP and NTS oscillators are both positive and comparable in magnitude, with the AP to NTS coupling around 30% larger. In our previous experimental investigation^30^ we concluded that the NTS had a negligible effect on the periodicity of the AP, since surgically removing one side of the NTS did not alter the AP period. We have shown here that this result is possible even when there are reciprocal interactions between the AP and the NTS (Fig. 4). This occurs because removing one side of the NTS ($K_{an} \rightarrow \frac{1}{2}K_{an}$) only reduces the total coupling by around 20%, which is similar to reducing $s = 1 \rightarrow 0.8$ in Fig. 3. Hence, the overall state of the system is relatively unaffected by the modification.

Communication of circadian phases between the AP and NTS likely involves action potential-dependent neuronal coupling. Functional neural projections from the AP to the NTS remain viable within our slice preparations^30,33,34,55^, and our previous experimental study indicates that the NTS is more responsive to AP stimulation during the night^30^ and that both structures exhibit daily variation in neuronal excitability^30,56^. Neural projections from the AP to the NTS are extensively documented^24,57,58^, and notably, noradrenalin can be co-localized with glutamate in AP axon terminals in the NTS^59,60^. It is currently unclear whether this or other AP-derived signals are recruited to communicate circadian phase, and future studies are necessary to resolve this. Further, the neurochemical phenotype of both AP and NTS oscillating cells is unknown. Both structures contain multiple cell types of differing functions, and identification of the key neurochemicals coexpressed with the molecular clock machinery will progress our understanding of both mechanism and function of DVC circadian rhythms. Further, dual-colour bioluminescent technology^61^ in combination with the modelling framework presented here, could illuminate the roles that individual cellular populations play in orchestrating DVC rhythmicity. Our results indicate that NTS-to-AP communication is also important for maintaining circadian phasing between the two structures. Anatomical evidence supports the possibility of this pathway^57,58,62^, however functional connectivity should be confirmed and the efferent NTS cell types should be identified.

We restricted our modelling to coarse-grained nuclei-wide rhythms, without treating the single-cell oscillations of the AP and NTS that we report in our earlier work^30^. The reason for this approach was due to insufficient cellular resolution in our 4Vep PER2::LUC images, and as such, we aimed to constrain our approach. Since our analysis indicates that the AP and NTS can synchronise independently of the 4Vep, it will be of interest to apply other techniques such as that employed by^63^ to the AP and NTS. In that study, individual cellular oscillations of the SCN subcompartments (core and shell) were modelled using the same phase oscillator framework as in the present study, and intraSCN-wide interactions emerge from many cellular interactions. Taking this approach and simulating single-cell oscillations could yield similar outcomes to those we present here, but the additional complexity provided by a large array of oscillators could aid in resolving why a phase lag is required in the whole-structure description. This is a limitation of a simple phase oscillator description and more mechanistically detailed models capturing the dynamics of intracellular clock gene expression may be necessary^64–66^.

A feature that could be included in a range of future models is the existence of both neuronal and non-neuronal couplings. Tetrodotoxin (TTX) blocks action potential-dependent communication, and its application has differential effects on the AP and NTS. The cellular oscillations within the NTS show more pronounced desynchrony compared to the AP following TTX application^30^. This indicates that the AP maintains its rhythmicity by utilizing action potential independent mechanisms, such as gap-junctions or that communication among non-neuronal cells. It is notable that both of these mechanisms are used by the SCN circadian pacemaker^67–69^. Using theoretical models to discriminate the role of these and other forms of intercellular communications will be useful in understanding this cell-cell coupling in these structures.

Identification of the physiological mechanisms that lead to a phase lag in the AP-NTS coupling is also an aim for future studies. We have used this parameter to tune the AP-NTS phase difference because realistic phase differences are unobtainable without it (see Supplementary Information). We estimate the phase lag to be 2.9 h, however, it is a phenomenological parameter and its biological significance remains to be determined. Currently, it represents an unknown in AP-NTS communication and it could be an emergent property of multiple non-linear oscillators. It is interesting that stronger AP-NTS coupling leads to larger a phase separation between the oscillators (Fig. 3), since this is the opposite of what others have found in coupled oscillator systems^51^. This emphasises that there are unknown processes underlying AP-NTS circadian coupling which require further investigation. An interesting possibility is that the glial barrier separating the NTS from the AP can limit communication between these structures. Experimentally, we showed PER2::LUC expression is rhythmic in this area and that the barrier is more permeable at night than during the day^30^. Therefore, this barrier is potentially a fourth DVC oscillator that interfaces with both the AP and NTS.

Our model fitting process indicates phase communication is negligible from the AP to the 4Vep and significantly stronger from the 4Vep to the AP, which is reflected by the parameters in Fig. 2E. Our analysis suggests this AP-4Vep topology because the frequency of synchronised AP-4Vep clusters is much closer to the intrinsic frequency of the 4Vep than the AP. However, the intrinsic properties of the 4Vep are not easily examined due to its small size and position between the other oscillators. Here we have estimated its intrinsic frequency from experiments in which the 4Vep phase dynamics resemble an uncoupled oscillator. Future studies should aim to culture the 4Vep oscillator separately from the rest of the DVC, first to assess the autonomy of the oscillator and secondly, to calculate its true intrinsic frequency.

It is currently not known how the ependymal cells in the 4Vep communicate with the AP. Unlike neurons, ependymal cells do not have axonal projections and it is unknown whether they secrete factors locally within the DVC to influence the other circadian oscillators. Despite the uncertainty, it is interesting to speculate that the unidirectionality arises as a consequence of the 4Vep cells’ access to the cerebral spinal fluid, which contains oscillating concentrations of signalling factors secreted by other circadian oscillators^41^. This could position the 4Vep as a receiver and integrator of circadian signals, relaying the rhythmic output of the rostrally positioned hypothalamic nuclei^37,70^ to the dorsal vagal complex.

The DVC serves as a site for integrating central (brain) and peripheral physiology. It is implicated in many processes including regulation of food intake and control of heart and lung activity^71–73^ which vary over the day-night cycle. Circadian oscillations within the DVC are altered in unhealthy metabolic or cardiovascular states^26,27,29^, and the relationship between oscillator phases may encode functionally relevant information. Here, we have provided a simple explanation for how phase difference patterns are maintained between the three DVC oscillators. With relatively simple network coupling, a phase model tuned to the DVC can reproduce the phase difference dynamics observed from wavelet analysis of rhythms in PER2::LUC expression. Further experimental and modelling work will be key to illuminating how intrinsic circadian oscillators interact with recurrent input from the periphery to shape the activity of the DVC across 24 h.

## Methods

### Animals

This study is based on a reanalysis of previously published results^30^ supplemented with new experimental data using laboratory animals. Here, we used nine PERIOD2::LUCIFERASE (PER2::LUC) mice of both sexes, aged from 2 to 4 months. Mice were on a C57Bl6j background and were generated from breeding stock kindly supplied to HDP by Dr. Pat Nolan of MRC Harwell UK. These mice were originally created and generously gifted to the scientific community by the laboratory of Dr. J. S. Takahashi^74^. All mice were bred in the Animal Services Unit at the University of Bristol under standard 12:12 h light-dark conditions with ad libitum access to water and food. Experiments were performed in accordance with the UK Animal (Scientific Procedures) Act 1986 and were approved by the local AWERB committee. The study was conducted consistent with ARRIVE 2.0 guidelines^75^.

### Bioluminescence recordings

#### Tissue preparation

Nine adult PER2::LUC mice were killed by approved Schedule 1 UK Animal (Scientific Procedures) Act 1986 protocol. Briefly, animals were terminally anaesthetised with the intra-peritoneal injection of sodium pentobarbital (80 mg/kg) and decapitated. Brains were immediately removed and cut into 250 μm thick coronal brainstem slices containing the intermediate part of the DVC (at the level of the AP) in the ice-cold Hank’s Balances Salt Solution (HBSS, Sigma, Germany) supplemented with 1 mg/ml penicillin-streptomycin (Sigma, Germany) and 0.01 M HEPES (Sigma) using a vibroslicer (Camden Instruments, UK). Following, the DVC was manually dissected with the scalpel and placed on the 30 mm Millicell cell culture inserts (Merck, Germany) in glass coverslip sealed Fluorodish culture dishes (World Precision Instruments Ltd., USA) with sterile culture medium (Dulbecco’s Modified Eagle’s Medium; DMEM, Sigma) supplemented with 0.1 mM luciferin (Promega, USA), B27 (Gibco Invitrogen Ltd, USA), 1 mg/ml penicillin-streptomycin (Gibco Invitrogen Ltd), 10 mM HEPES (Sigma) and 3.5 g/L d-glucose (Sigma). For the AP-disconnection study, the APs were totally cut off the DVC using the surgical blade and further placed on the same culture membrane near the remaining parts of the DVC.

#### Data acquisition and initial analysis

Images were taken with the Olympus Luminoview LV200 (Olympus, Japan) fitted with a cooled Hamamatsu ImageEM C900-13 EM-CCD camera and a $20 \times 0.4$ NA Plan Apo objective (Olympus, Japan) on a heated stage kept at 37 °C. Gain and exposure time was constant throughout the recordings. For these experiments we used 30 min exposure time, but to match it with our previous data^30^, we further binned the datapoints into 1 h epochs. Raw images were initially analysed in FIJI (ImageJ, NIH, USA) using a polygon region of interest tool for the whole brain areas.

### Wavelet analysis

Raw PER2::LUC time series were detrended using a sinc filter (cutoff frequency of 48 h) and time-frequency spectrum’s were calculated by a continuous wavelet transform using the Python package pyBOAT^35,76^ (minPer = 10 h, maxPer = 48 h, numberOfPers = 101, ridgeThreshold = 0).

### Analysis of phase difference stability

Phase difference traces were used to construct 15 min-binned histograms (PD measured in minutes). A histogram with a unimodal peak suggests that the PD remained at the value of the peak for a considerable portion of the recording. The prominence of the maximum peak in a histogram was assessed using the metric $y=\max _{i} \{h_i\}/\sum _{i=0}^N h_i$, where $\{h_1, ..., h_N\}$ are the values of the *N* bins. High values of *y* correspond to a prominent peak whereas low values indicate a more uniform distribution of phases. See Supplementary Fig. S5 for more details.

### Analysis of constant phase difference dynamics

A state of constant PD between any two oscillators is identified if the rate of change of PD is less than 0.01 rad/h ($| d\theta _{ij}/dt| < 0.01$) for at least 24 h. The constant PD between two oscillators, $\theta _{ij}^*$ (Fig. 2C), is calculated as their average PD whilst this condition is met. The collective frequency of two oscillators with a constant PD, $\tau _{ij}^*$ (Fig. 2D), is defined as the mean frequency between the two oscillators whilst their PD is constant. Examples of regions of constant PD, and their corresponding collective frequency are shown in Fig. 2A, Supplementary Figs. S3 and S4.

### Derivation of mathematical model

The PER2::LUC signals are a bioluminescent glimpse into the complexity of the molecular circadian clock. In full, the clock mechanism consists of multiple genes and protein interactions, the dynamics of which can be assumed to map onto a limit cycle attractor. Following the phase reduction methods established by Winfree^77^, the dynamics of a single limit cycle variable, such as PER2, can be investigated in isolation by mapping the dynamics around the limit cycle to the motion around the unit circle^78–81^. This method describes a multi-dimensional limit cycle using a single phase variable, which we use here to model the phase of PER2::LUC oscillations.

Each of the nuclei-wide PER2::LUC bioluminescent oscillations from the AP, the NTS and the 4Vep are described by individual phase variables. The NTS is effectively bilateral in the coronal sections that our data is from (these bilateral structures fuse together caudle of the AP). Under the assumption of identical bilateral oscillations and symmetrical interactions between the AP and the two unilateral structures, we model the NTS as a single oscillator. In the absence of interactions, an oscillator will evolve at its intrinsic frequency, $\omega _i$, which is described by the ordinary differential equation (ODE): $d\theta _i/dt=\omega _i$. The intrinsic frequency is related to the intrinsic period, $\tau _i$, by $\omega _i=2\pi /\tau _i$. Interactions alter the evolution of phases, and hence frequencies (since frequencies are described by $\omega _i(t)=d\theta _i/dt$), and they are usually assumed to depend upon phase differences, $\theta _{ij}=\theta _i-\theta _j \in (-12, 12]$ h. The functional form of the interaction ($\Gamma (x)$) is largely free to choose, however, a realistic function should be odd ($\Gamma (x-)=\Gamma (x)$) and periodic ($\Gamma (x+2\pi )=\Gamma (x)$). We choose the conventional $\sin (x)$ interaction function, which is the dominant term in the expansion of $\Gamma (x)$, giving us a Kuramoto model^36^. This leads to the following set of ODEs for the phases of the PER2::LUC oscillations from the AP ($\theta _a$), the NTS ($\theta _n$) and the 4Vep ($\theta _v$)

2 $$\begin{aligned} \begin{aligned} \dot{\theta _a}&= \omega _a + K_{an}\sin (\theta _n - \theta _a + \gamma ) + K_{av}\sin (\theta _v - \theta _a) \\ \dot{\theta _n}&= \omega _n + K_{na}\sin (\theta _a - \theta _n - \gamma ) + K_{nv}\sin (\theta _v - \theta _n) \\ \dot{\theta _v}&= \omega _v + K_{va}\sin (\theta _a - \theta _v) + K_{vn}\sin (\theta _n - \theta _v), \\ \end{aligned} \end{aligned}$$

where $\dot{\theta }_i = d\theta _i / dt$ and phases are 24 h periodic variables defined between − 12 and 12 h. The strength of an interaction from oscillator *i* to oscillator *j* is modulated by the scalar parameter $K_{ji} \in \mathbb {R}$, and the set of all coupling parameters describes an interaction network. Coupling can be repulsive, with $K_{ij}<0$, or attractive, with $K_{ij}>0$. Both forms of coupling promote synchronisation, in the sense of the equivalence of frequencies, with attractive coupling promoting in-phase synchrony ($\theta _{ij}\rightarrow 0$) and repulsive coupling promoting anti-phase synchrony ($\theta _{ij}\rightarrow 12$ h). The AN phase lag, $\gamma$, is a parameter that breaks the symmetry of AP-NTS interactions thereby allowing non-zero interactions between the oscillators when they are perfectly in phase. It is analogous to including a $\cos (x)$ component in the expansion of $\Gamma (x)$ and we use it here to tune steady-state AN-PD values. The justification for including $\gamma$ can be found in the Supplementary Information.

### Estimation of model coupling parameters

to estimate the coupling parameters in each of the sub-systems individually describing the AN-PD and the AV-PD, we focused on the top 5 most stable PD datasets that were unimodal in their PD distribution (AN-PD: experiments 3, 4, 5, 7 and 12; AV-PD: experiments 3, 4, 7, 8 and 9; see Supplementary Fig. S5) Some AN-PD trajectories that scored highly for stability were also multistable, having two regions of nearly constant PD. We excluded these traces from model fitting because it was ambiguous which PD plateau should be considered the steady state, and we also find that multiple plateaus arise in model simulations with decaying coupling, but not with constant coupling (Fig. 4C; black curve). The assumption is that the most stable data with a single PD plateau are simulated by a model above the synchrony threshold. This allows us to compare the steady state of the model, where variables are constant, with the approximately constant states in our data.

#### AP-4Vep coupling

An AP-4Vep phase model consists of the equations

3 $$\begin{aligned} \dot{\theta }_{av}&= \omega _{av} - \tilde{K}_{av}\sin \theta _{av} \end{aligned}$$

4 $$\begin{aligned} \Omega _{av}(t)&= \langle \omega \rangle _{av} + \frac{\Delta K_{va}}{2} \sin \theta _{av}, \end{aligned}$$

where $\Omega _{ij} = \dot{\langle \theta \rangle }_{ij} = \frac{1}{2}(\dot{\theta }_i + \dot{\theta }_j)$ is the mean frequency of oscillators *i* and *j* and $\langle \cdot \rangle _{ij}$ denotes the mean. It is simple to compare this model to the AV-PD dynamics to estimate coupling parameters. The steady state of Eq. (3) is given by $\theta _{av}^* = \arcsin \left( \omega _{av}/\tilde{K}_{av}\right)$, hence $\tilde{K}_{av}=K_{av}+K_{va}$ can be calculated since we know the constant AV-PD ($\theta _{av}^*$) and the initial frequency detunings ($\omega _{av}$). Equation (4) for the mean frequency of the AP-4Vep system can be rearranged to calculate the difference in the coupling terms $\Delta K_{av}= K_{va} -K_{av} = 2\left( \Omega _{av}^* - \langle \omega \rangle _{av} \right) / \sin \theta _{av}^*$, where $\Omega _{av}^*$ is the mean frequency of the two oscillators whilst their PD is constant.

#### AP-NTS coupling

 Estimating the AP-NTS coupling parameters and phase lag is less straightforward because the extra parameter results in degeneracy: different combinations of $(\tilde{K}_{an}, \gamma )$ lead to the same steady state $\theta _{an}^*$. To identify the parameter set that best describes the data, an extra constraint, the time taken for perturbations to decay, is used. The PD and mean frequency of AP-NTS system is given by

5 $$\begin{aligned} \dot{\theta }_{an}&= \omega _{an} - \tilde{K}_{an}\sin (\theta _{an}-\gamma ) \end{aligned}$$

6 $$\begin{aligned} \Omega _{an}(t)&= \langle \omega \rangle _{an} + \frac{\Delta K_{na}}{2} \sin (\theta _{an}-\gamma ), \end{aligned}$$

Linear stability analysis tells us that a small perturbation from the steady state will exponentially decay at a rate $\lambda = -\tilde{K}_{an}\cos (\theta _{an}^*-\gamma )$. Combining this with the expression for the steady state, $\sin (\theta _{an}^*-\gamma )=\omega _{an}/\tilde{K}_{an}$, gives an expression for $\tilde{K}_{an}$ in terms of a linear decay rate and initial detuning: $\tilde{K}_{an} = \sqrt{\lambda ^2 + \omega _{an}^2}$. To calculate linear decay rates from the most stable subset of data, we fit exponential curves ($y=ae^{\lambda t} +b$) to the AN-PD time series when the PD is sufficiently close to the steady state (see Supplementary Fig. S4A). With $\tilde{K}_{an}$ known, the phase lag $\gamma$ can be calculated using the expression for the steady-state of (5). The equation for the collective phase gives an expression for the difference in coupling parameters, $\Delta K_{an} = 2\left( \Omega _{an}^* - \langle \omega \rangle _{an} \right) / \sin (\theta _{av}^*-\gamma )$, which allows us to estimate the coupling constants $K_{an}$ and $K_{na}$ (Fig. 2E). Supplementary Figs. S3 and S4 show how the two-oscillator models compare to individual PD data.

## Model simulations

The systems of ODEs within this article were solved in Python using the SciPy function *solve_ivp* with a Runge-Kutta (order 5/4) method and $rtol=10^{-3}$ and $atol=10^{-6}$. Bifurcation analysis was performed using the MATLAB numerical continuation software MatCont^82^.

## Supplementary Information

Supplementary Information.

## Data Availability

All data used in this article can be obtained upon request from the corresponding author.
