# Supplementary material for: A new phase model of the spatiotemporal relationships between three circadian oscillators in the brainstem
Source: Sci Rep. 2023 Apr 4;13:5480. doi: 10.1038/s41598-023-32315-y (PMC10073201; doi:10.1038/s41598-023-32315-y)
Supplement: Supplementary file 1 — Supplementary Information. [file 41598_2023_32315_MOESM1_ESM.pdf]

Supplementary Information for *Ahern et al. A new phase model of the spatiotemporal relationships between three circadian oscillators in the brainstem (2023)*

Phase difference dynamics of the hindbrain circadian network

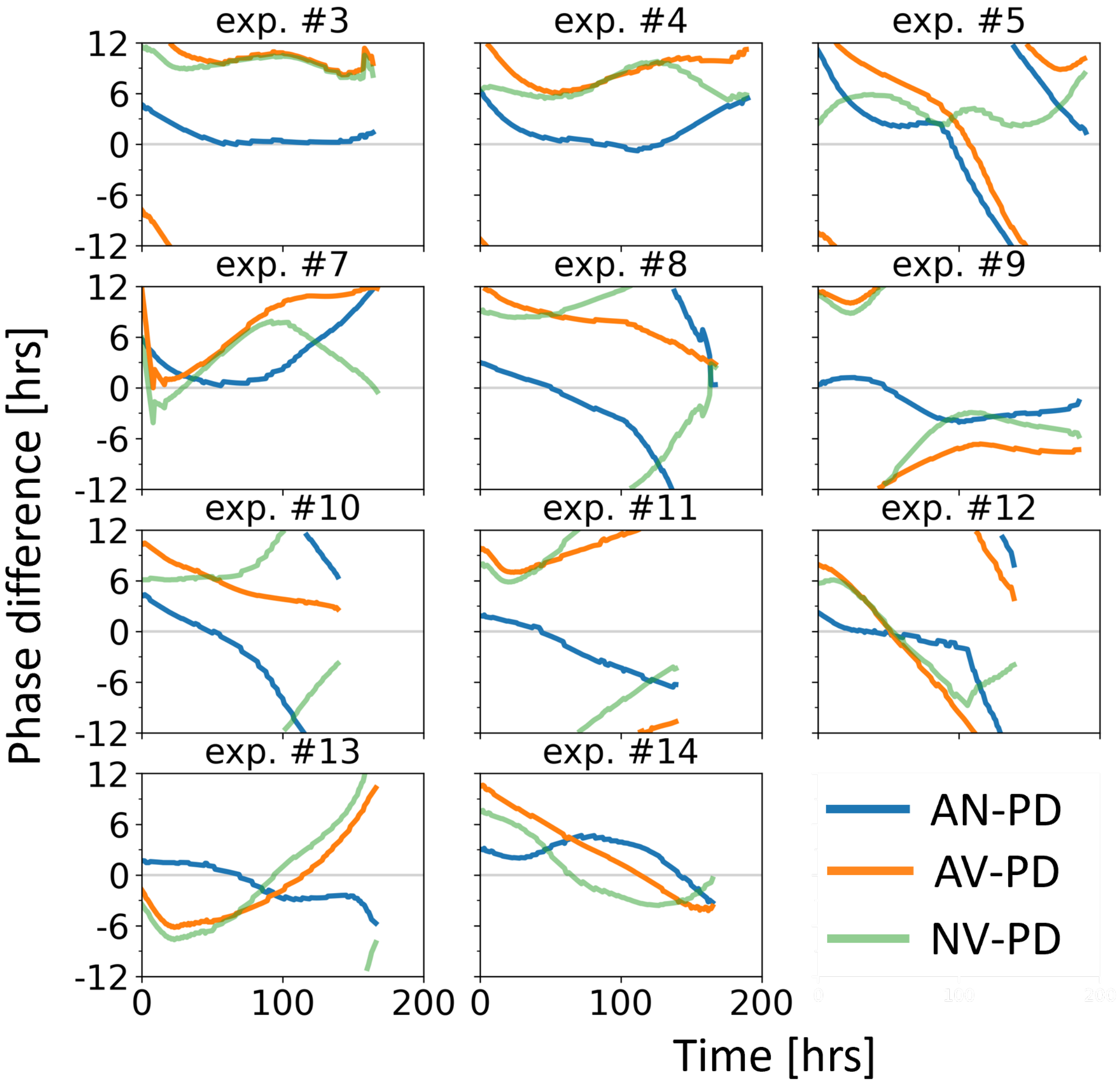

Supplementary Figure S1. Phase difference dynamics from all 11 PER2::LUC bioluminesence imaging experiments. The phase difference is plotted in hours [hrs] and is periodic such that $PD = 12$ is equivalent to $PD = -12$. The phase differences are obtained using wavelet deconvolution, as described in the Methods section of the main text.

Period dynamics of the hindbrain circadian network

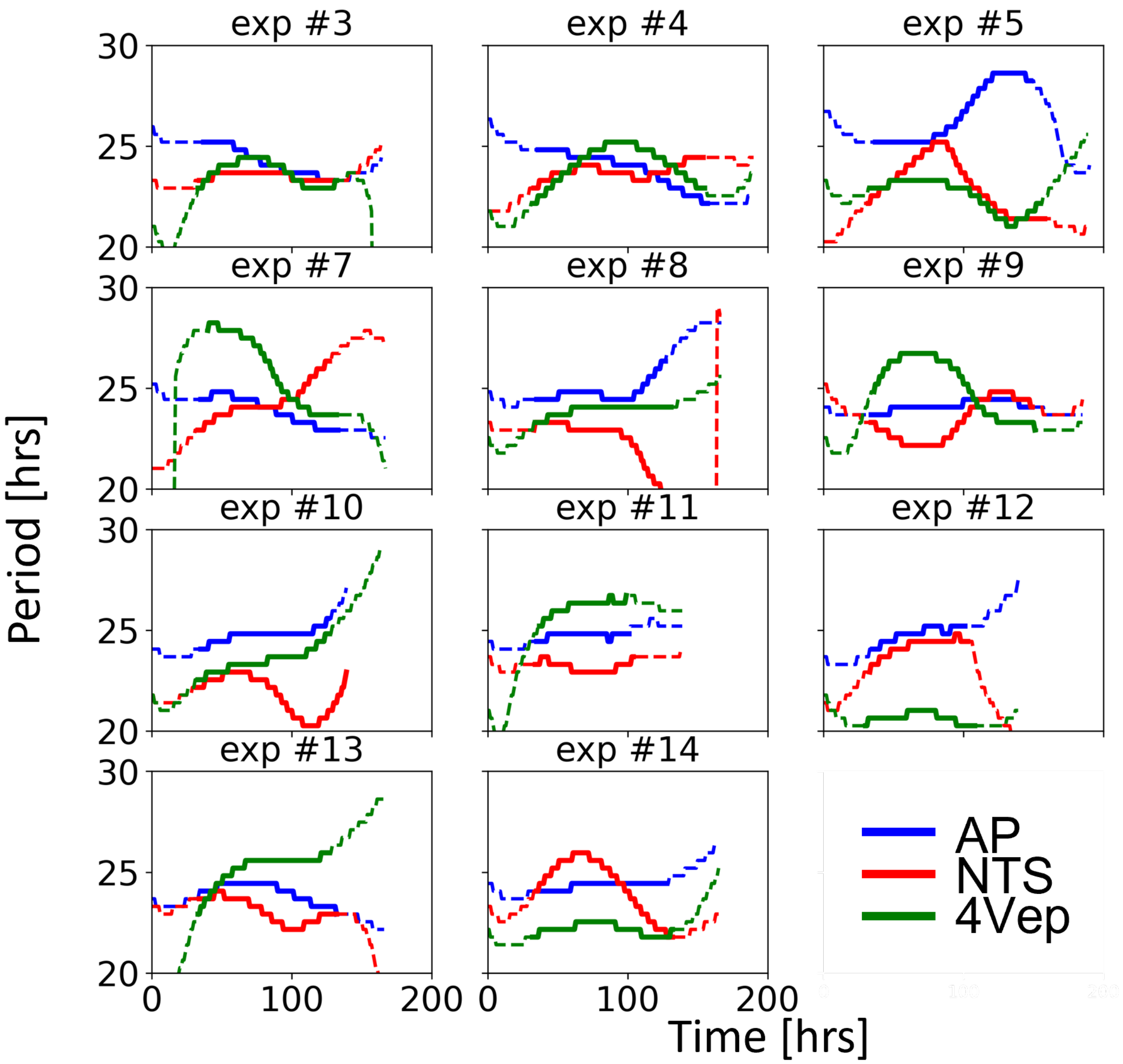

Supplementary Figure S2. Period dynamics from all 11 PER2::LUC bioluminesence imaging experiments. The dotted lines are dynamics within the 'cone of influence' (see Monke et al. (2020)), which are subject to boundary effects.

AP-4Vep PD analysis and estimation of coupling parameters

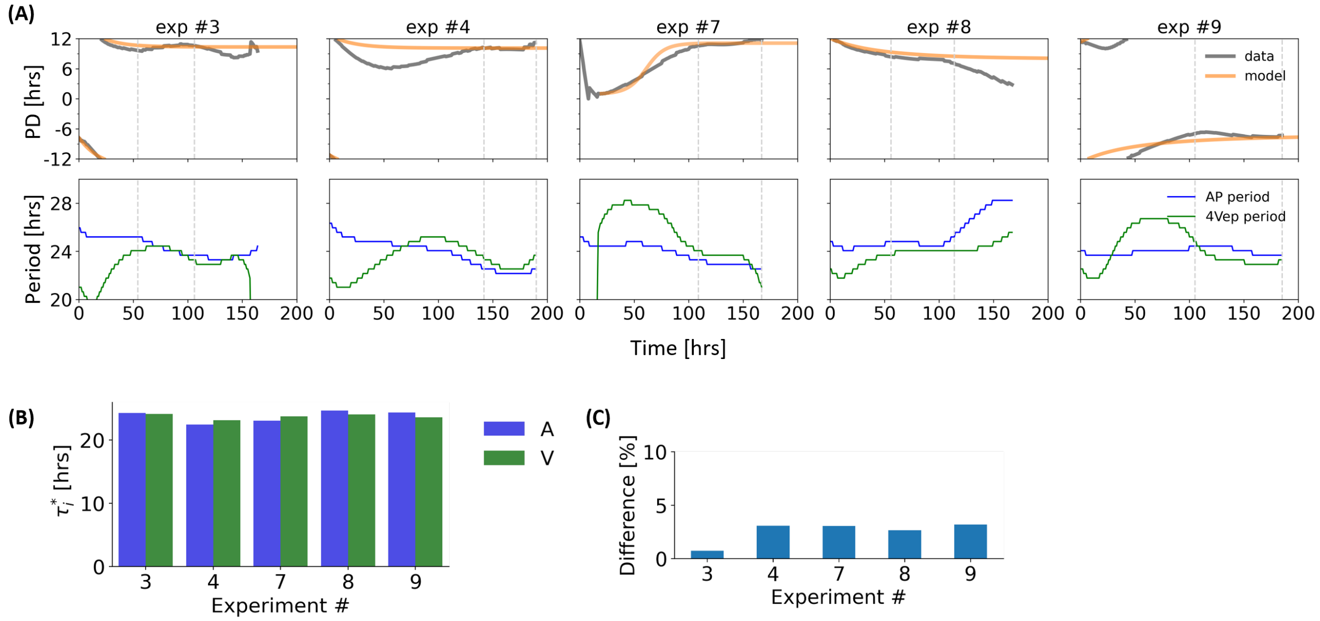

Supplementary Figure S3. AP-4Vep PD analysis and estimation of coupling parameters. **(A)** The AV-PD dynamics of the five experiments that were selected to fit the AP-4Vep phase oscillator model. Each data trajectory (grey) has a period of constant PD between the vertical dashed lines, defined such that $|d\theta_{av}/dt| < 0.01$. After estimating the parameters of an AP-4Vep coupled oscillator model, the simulated trajectory (orange) matches well with the data. During an epoch of constant PD, the two oscillators are approximately at the same frequency, indicated by nearly identical period dynamics in **(B)**. The average period over this epoch for each oscillator in all five experiments is plotted in **(C)**. The difference between the oscillator's average period within this epoch is plotted in **(D)**. The small differences suggest that the oscillators are approximately at the same period, hence their collective period is calculated as the mean period between the oscillators throughout this epoch.

AP-NTS PD analysis and estimation of coupling parameters

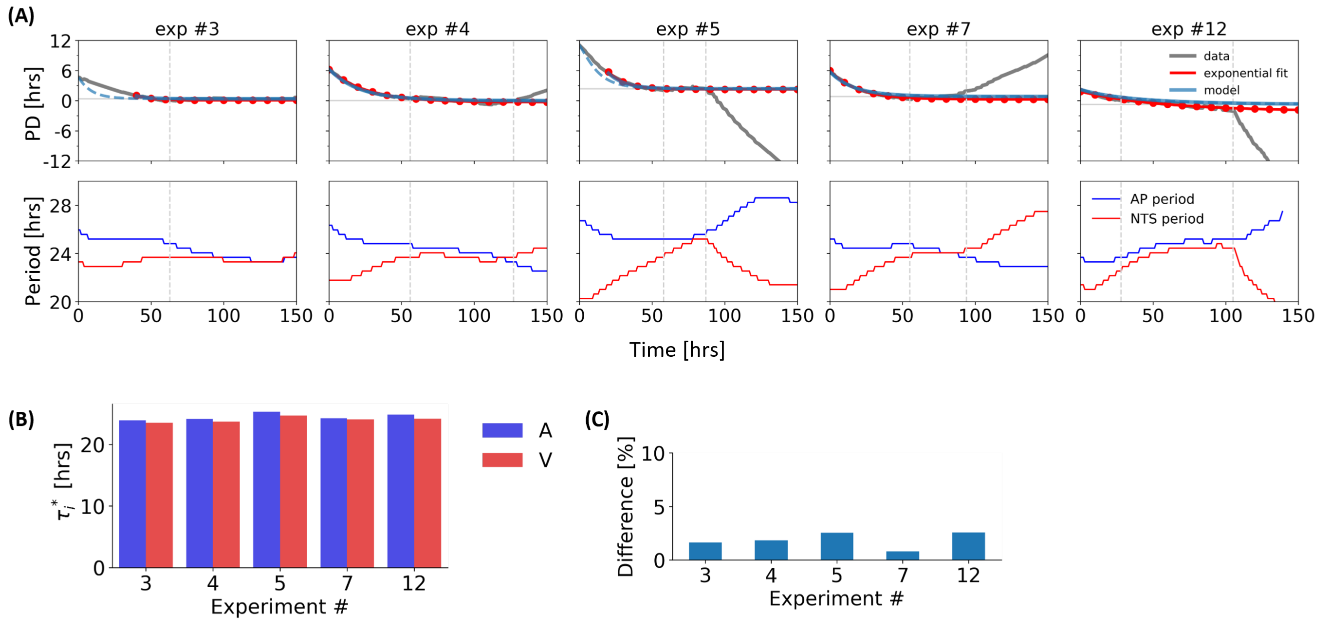

Supplementary Figure S4. AP-NTS PD analysis and estimation of coupling parameters. **(A)** The AN-PD dynamics of the five experiments that were selected to fit the AP-NTS phase oscillator model. Each data trajectory (grey) has a period of constant PD between the vertical dashed lines, defined such that $|d\theta_{an}/dt| < 0.01$. After estimating the parameters of an AP-NTS coupled oscillator model, the simulated trajectory (blue) matches well with the data. To estimate all the parameters of the AP-NTS model, the linear decay rate of the PD data was estimated by fitting an exponential curve (red dots) to the PD trajectory when it is sufficiently close to its constant PD state. During an epoch of constant PD, the two oscillators are approximately at the same frequency, indicated by nearly identical period dynamics in **(B)**. The average period over this epoch for each oscillator in all five experiments is plotted in **(C)**. The difference between the oscillator's average period within this epoch is plotted in **(D)**. The small differences suggest that the oscillators are approximately at the same period, hence their collective period is calculated as the mean period between the oscillators throughout this epoch.

Analysis of the stability of phase differences

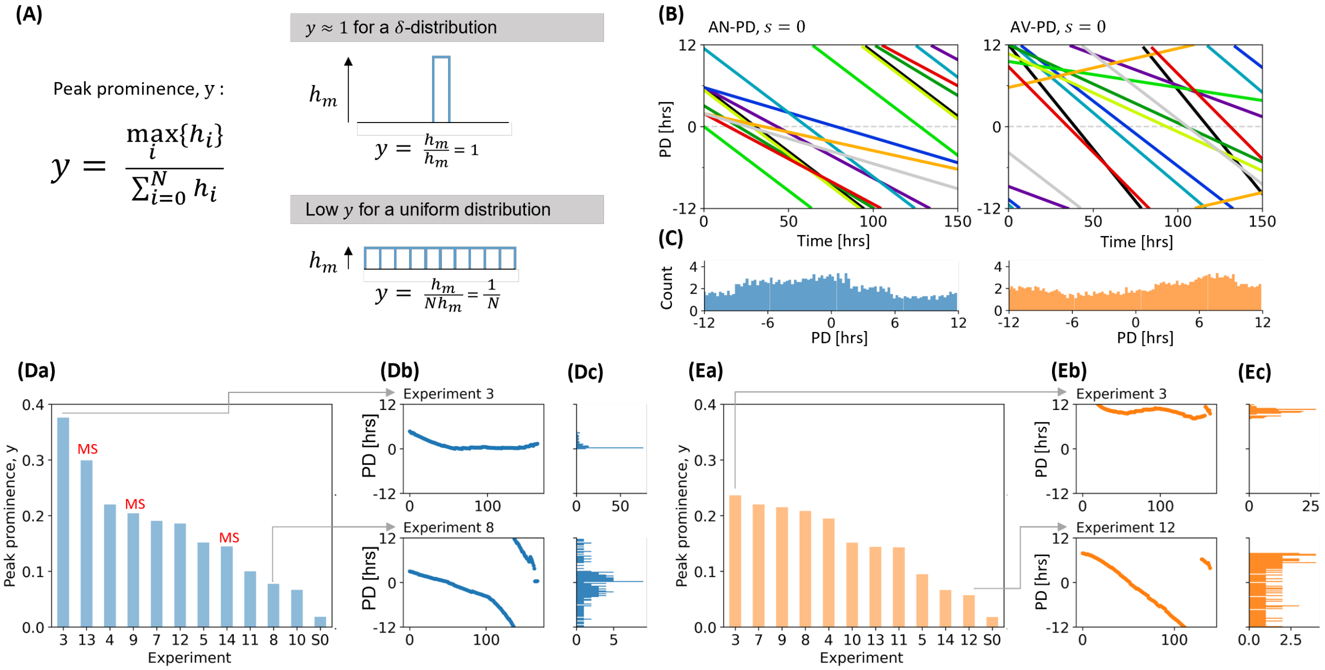

Supplementary Figure S5. The prominence of a peak in the histogram of phase differences is an indication of the stability of the PD time series. The prominence is measured using the expression in (**A**), which is 1 for a histogram with all its weight within one bin (the d-distribution) and close to zero ($1/N$, where N is the number of bins) when the distribution is uniform. This metric provides a measure of the stability of a PD trajectory. Simulations of the model system (1) with realistic periods and initial conditions (the same used in the simulations of Figure 4C and D) and zero coupling ($s = 0$) are shown in (**B**). PD trajectories of uncoupled oscillators are linear and display no tendency to remain at a particular PD value, which is indicated in the histograms in (**C**) (averaged over all simulations in (B)). Such distributions are nearly uniform and have a low y value, indicated as 'S0' in (Da) and (Ea). In (**Da**) and (**Ea**), the peak prominence is displayed for all 11 experiments, and example time series and histograms (15min-binned) are shown in parts b and c respectively. Three AN-PD traces are multistable (red 'MS'), as shown by the trajectories in Supplementary Figure S1 and their PD histograms displayed bimodality. Despite their high score for stability, these traces could not be used to fit the model since it was ambiguous which near-constant PD should be considered the as the steady-state. Furthermore, similar occurrences of multiple constant PD regions arise in simulations with slowly decaying coupling (Figure 4C; black curve), hence this could be an effect of reducing the oscillators coupling.

Additional mathematical details

The NTS is modelled as a single oscillator

For demonstrating the assumptions underlying the description of the whole NTS PER2::LUC rhythm by a single phase variable, we shall consider an AP-NTS system. Consider an AP (θ_a) oscillator connected to two NTS oscillators ($\theta_{n_1}, \theta_{n_2}$). The phase dynamics are given by

$$\begin{aligned}
\dot{\theta}_a &= \omega_a + K_{an_1}\sin(\theta_{n_1} - \theta_a) + K_{an_2}\sin(\theta_{n_2} - \theta_a) \\
\dot{\theta}_{n_1} &= \omega_{n_1} + K_{n_1 a}\sin(\theta_a - \theta_{n_1}) + K_{n_1 n_2}\sin(\theta_{n_2} - \theta_{n_1}) \\
\dot{\theta}_{n_2} &= \omega_{n_2} + K_{n_2 a}\sin(\theta_a - \theta_{n_2}) + K_{n_2 n_1}\sin(\theta_{n_1} - \theta_{n_2}),
\end{aligned} \tag{1}$$

First, we assume that the oscillatory signals from both NTS are identical, such that $\omega_{n_1} = \omega_{n_2} = \omega_n$ and $\theta_{n_1} = \theta_{n_2} = \theta_n$. Next, we assume that AP-NTS interactions are symmetrical across hemispheres, such that $K_{n_1 a} = K_{n_2 a} = K_{na}$ and $K_{an_1} = K_{an_2 1} = K_{an}$. This results in

$$\begin{aligned}
\dot{\theta}_a &= \omega_a + 2K_{an}\sin(\theta_n - \theta_a) \\
\dot{\theta}_n &= \omega_n + K_{na}\sin(\theta_a - \theta_n) \\
\dot{\theta}_n &= \omega_n + K_{na}\sin(\theta_a - \theta_n).
\end{aligned} \tag{2}$$

Clearly, these assumptions result in either NTS being described by the same equation, hence it makes sense only to study one variable to represent the NTS oscillations.

This mathematical consideration also motivates the protocol we use for our simulated NTS disconnect experiment. Disconnecting one NTS results in removing AP-NTS interactions from one hemisphere, $K_{an_2} = K_{n_2 a} = 0$, and removing any possible communication between either side of the NTS, $K_{n_1 n_2} = K_{n_2 n_1} = 0$. Applying this disconnection to equation 1, whilst assuming that the intrinsic properties are unchanged gives

$$\begin{aligned}
\dot{\theta}_a &= \omega_a + K_{an_1}\sin(\theta_{n_1} - \theta_a) \\
\dot{\theta}_{n_1} &= \omega_n + K_{n_1 a}\sin(\theta_a - \theta_{n_1}) \\
\dot{\theta}_{n_2} &= \omega_n.
\end{aligned} \tag{3}$$

Under the assumption of bilaterally symmetric AP-NTS coupling, $K_{an_1} = K_{an_2} = K_{an}$, we see that removing one NTS results in a reduction of the NTS to AP coupling ($K_{an} = K_{an_1}$) by one half (compare the first equations of systems 2 and 3).

A phase lag, γ, is required in the AP-NTS coupling term

To motivate the inclusion of the phase lag, γ, in the AP-NTS coupling term consider an isolated AP-NTS system, the phase difference of which is given by

$$\dot{\theta}_{an} = \omega_{an} - \tilde{K}_{an} \sin \theta_{an}, \tag{4}$$

where $\tilde{K}_{an} = K_{an} + K_{na}$, and $\omega_{an} = \omega_a - \omega_n < 0$. The steady-state solution, θ^*_{an}, and the eigenvalue at this solution, λ, is given by

$$\sin \theta^*_{an} = \omega_{an}/\tilde{K}_{an} \quad \text{and} \quad \lambda = -\tilde{K}_{an} \cos \theta^*_{an}, \tag{5}$$

respectively. In our experiments, we observe small phase differences around zero between the AP and NTS, and so we want a stable (biologically observable) solution to be small, which we define as $\theta^*_{an} \in [6,6]$ hrs ($[-\pi/2, \pi/2]$ rad). For this solution to be stable we require $\lambda < 0$, and since $\cos \theta^*_{an} > 0$ this implies that $\tilde{K}_{an} > 0$. Hence for small phase differences to be stable, we require that the sum of the coupling is positive. However, this immediately leads to a problem since for $\tilde{K}_{an} > 0$, equation 4 only allows positive solutions; the minimum phase difference possible, in the limit of infinite coupling, is zero. This does not agree with our observations of constant phase differences below zero (see Supplementary Figure S1, experiments 9, 12 and 13). Negative, stable solutions would be possible if there were an overlap in the intrinsic frequency distributions of the AP and the NTS, but our data reveals that this is not the case. We include a phase lag into the AP-NTS interaction terms to allow phase difference states around zero to be stable.
